# Cutaneous adverse events associated with the efficacy and benefit of immune checkpoint inhibitors: a systematic review and meta-analysis

**DOI:** 10.3389/fonc.2025.1689519

**Published:** 2025-11-07

**Authors:** Li Zhang, Yunge Gao

**Affiliations:** The People’s Liberation Army (PLA) Strategic Support Force Characteristic Medical Center (The 306th Hospital of PLA), Beijing, China

**Keywords:** cutaneous immune-related adverse event, immune toxicity, immune checkpoint inhibitor, efficacy, prognosis

## Abstract

**Objective:**

Cutaneous immune-related adverse events (cirAEs) are associated with improved survival in tumor patients undergoing immune checkpoint inhibitors (ICIs) therapy, while inconsistent evidence has been reported across tumor subtypes. This study aimed to evaluate the association of cirAEs and their subtypes with cancer prognosis.

**Design:**

Systematic review and meta-analysis.

**Methods:**

Four databases including Cochrane Library, Embase, PubMed and Web of Science were searched for original publications from inception to April 30, 2024. A meta-analysis was carried out for overall survival (OS) and progression-free survival (PFS) of patients, and pooled hazard ratios (HRs) with 95% confidential intervals (CIs) were calculated. Subgroup analyses involving cirAEs subtype, cancer type, ICIs type, geographic region of patients, and study quality were performed.

**Results:**

Forty-five studies comprising a total of 26817 patients with ICIs treatment were included in the study. The occurrence of cirAEs was associated with prolonged OS (HR, 0.54 [95%CI, 0.46-0.63]; *P* < 0.001) and PFS (HR, 0.51 [95%CI, 0.43-0.60]; *P* < 0.001). Favorable survival was observed in patients with vitiligo, with the most pronounced OS (HR, 0.23 [95%CI, 0.18-0.33]; *P* < 0.001) and PFS (HR, 0.28 [95%CI, 0.21-0.38]; *P* < 0.001). Similarly, the prolonged OS (HR, 0.69 [95%CI, 0.64-0.74]; *P* < 0.001) and PFS (HR, 0.69 [95%CI, 0.58-0.82]; *P* < 0.001) in patients with rash. Significant benefit in OS were also observed in drug hypersensitivity or eruption, eczematous, lichenoid or lichen planus-like lesion, pruritus and psoriasis, but not in bullous pemphigoid, maculopapular and mucositis. Favorable survival was observed in patients with RCC, NSCLC and MM, with the most pronounced OS (HR, 0.22 [95%CI, 0.08-0.59]; *P* = 0.002) and PFS (HR, 0.22 [95%CI, 0.11-0.43]; *P* < 0.001) for RCC patients; and only OS benefit in HNSCC (HR, 0.64 [95%CI, 0.42-0.99]; *P* = 0.04). Subgroup analyses involving geographic region and study quality showed consistent results with the overall estimate confirming robustness.

**Conclusions:**

The occurrence of cirAEs, especially in vitiligo and rash, predicted a significant survival benefits among tumor patients receiving ICIs therapy, especially in MM, RCC and NSCLC.

## Introduction

Over the past decade, immune checkpoint inhibitors (ICIs) have revolutionized the cancer therapeutic landscape for various advanced or metastatic malignancies (1–3), leading to improved tumor response and prognosis. These agents target immune checkpoints like cytotoxic T-lymphocyte-associated protein 4 (CTLA-4) and programmed cell death 1 or its ligand (PD-1/PD-L1), disrupting immunosuppressive pathways and immune homeostasis critical for cancer progression (4–6). ICIs now emerge as one of the main pillars of oncologic therapy, serving as the first-line treatments (4, 7).

As ICI therapy expand to multiple cancers, including melanoma (MM), non-small cell lung cancer (NSCLC), head and neck squamous cell carcinoma (HNSCC), renal cell cancer (RCC), and other solid/hematologic malignancies (3, 8–10), they may induce immune-related toxicity manifested as immune-related adverse event (irAE), which have become a key challenge. The excessive immune activation by ICIs may lead to the formation of autoantibodies in peripheral tissues and excessive off-tumor inflammation and autoimmunity (3, 10), which can subsequently affect multiple organ systems at any time during or after ICI therapy (9, 11). Growing evidences links specific irAEs (particularly cutaneous and endocrine events) to improved overall survival (OS) across solid tumors (12–15), offering potential prognostic insights for immunotherapy (16, 17).

Cutaneous irAEs (cirAEs) are the most common and earliest-onset irAEs observed in clinical practice with a broad spectrum of manifestations affecting approximately 20%-60% of subjects, and serve as potential biomarkers for treatment efficacy (6, 18). The current lack of systemically dermatologic terminology raises an urgent need for both oncologists and dermatologists to gain familiarity with cirAEs and their clinical impact (18). Previous studies have demonstrated associations between the incidence of cirAEs and its subtypes with improved prognosis across various ICI-treated malignancies, and cirAEs involving several rare manifestations are positively associated with improved clinical outcomes in cancer patients (13, 14, 19–21). Advanced urothelial cancer (UC) patients with cirAEs showed significantly prolonged OS, progression-free survival (PFS), and clinical benefit (CB), and irAEs could be potential biomarkers for UC and RCC (10, 15, 22). Similarly, favorable prognoses are observed in cutaneous squamous cell carcinoma and MM cohorts (12). Both vitiligo and non-vitiligo cirAEs correlate with superior OS in MM and pan-cancer settings (13, 14). Notably, compared with patients presenting single cirAEs, those manifesting multiple cirAEs showed better tumor responses across diverse cancers including melanoma, lung, gastrointestinal, head and neck, and other malignancies (15, 23). However, previous study stated that cirAEs might not a surrogate prognostic indicator, which are dose-independent and agent-specific immune reactions with the highest risk observed in CTLA-4 blockade (24).

To our knowledge, the relationship between cirAEs and survival outcomes in diverse clinical settings remains controversial (6, 24), regarding whether the association between cirAEs and survival is consistent across all cirAE subtypes and cancer types, and whether it holds after rigorous methodological adjustments (like landmark analysis). Besides, both oncologists and dermatologists critical need to gain familiarity with the cirAEs and its effect to clinical outcomes (14), given lack systemically dermatologic terms in relevant studies so far; meanwhile a large number of high-quality related studies have emerged. Therefore, we conducted a comprehensive systematic review and meta-analysis to explore the relationship between the incidence of cirAEs and their subtypes and the prognosis of patients receiving ICI therapy, which could be the largest meta-analysis on this topic, providing effective information for clinicians as well as novel insights for individualized and precise cancer therapy.

## Methods

### Search strategy and selection process

A systematic review and meta-analysis of the literature was conducted, and four databases (the Cochrane Library, Embase, PubMed and Web of Science) were searched to identify relevant articles from database inception to April 30, 2024. The search terms included: (immune related adverse event OR irAEs OR skin OR cutaneous adverse event OR cutaneous immune-related adverse event OR dermatological adverse event OR mucosal adverse event) AND (PD-1 OR programmed death receptor 1 OR PD-L1 OR programmed death-ligand 1 OR cytotoxic T lymphocyte associated protein 4 OR CTLA-4 OR immune-checkpoint inhibitor OR checkpoint blockade OR ICI) AND (prognosis OR survival OR benefit OR mortality OR efficacy OR outcome OR OS OR PFS) AND (cancer OR carcinoma OR tumor OR malignancy). Additional records were evaluated through manual search of the references from primary literatures and relevant reviews.

### Inclusion criteria

Eligible studies should meet the following inclusion criteria: (1) involved cancer patients receiving ICIs therapy; (2) published in any language with no restriction of publication year; (3) reported data on hazard ratios (HRs) with 95% confidence intervals (CIs) for OS and/or progression-free survival (PFS) comparing tumor patients with and without cirAEs; (4) employed prospective, retrospective, or clinical trial designs. Reviews, case reports, guidelines and editorials were excluded.

### Data extraction and collection and quality assessment

Two independent reviewers (L. Zhang and Y. Gao) screened records by title/abstract followed by full-text assessment. The same reviewers extracted data from each eligible study regarding study characteristics (author, publication year, geographic region, study design, cancer type, research center, follow-up duration), study population (sample size, sex distribution, median age), interventions (types of ICIs and therapy combination) and outcome data (number of cirAEs, specific cirAE types, analytical models, landmark analyses, HRs with their 95%CIs for OS and/or PFS). Study quality was assessed using the Newcastle-Ottawa Scale (NOS), with scores ranging from 0 to 9 based on three major domains: patient selection, comparability, and outcome assessment. Discrepancies were resolved through consensus-based discussion, and additional study information was obtained from the authors when necessary.

### Statistical analysis

In the study, both fixed-effects and random-effects meta-analyses were employed to evaluate the association between cirAEs and tumor prognosis by pooling HR with corresponding 95% CIs. HRs < 1 with 95% CIs not crossing 1 indicated favorable prognosis, whereas the converse suggested poor prognosis. Heterogeneity was evaluated by Cochran’s Q statistic and the I² statistic. Fixed-effects models were used for meta with no or low heterogeneity (*I²* < 50%, *P* > 0.05), and random-effects models were used for meta with moderate or high heterogeneity (*I²* ≥ 50%, *P* ≤ 0.05). To address potential guarantee-time bias, a landmark analysis was conducted at a predefined minimum 6-week time point. Only patients who were event-free and remained in the study at minimum 6 weeks were included in this validation analysis. Subgroup analyses were conducted when ≥ 2 datasets were available, stratified by cirAE subtypes, tumor type, immune checkpoint blockers (ICBs) regimen (monotherapy/combination therapy), study quality, and geographic region of patients. All subgroup analyses are considered exploratory and are interpreted with caution due to the increased risk of Type I error from multiple comparisons. Sensitivity analyses used the leave-one-out method, which involved sequential exclusion of individual studies, with concomitant application of fixed-effects and random-effects models to evaluate robustness of pooled effect estimates. Publication bias was evaluated via funnel plots, supplemented by Begg’s and Egger’s tests (two-tailed *P* < 0.05 indicating statistically significance). Trim-and-fill method was implemented to adjust for potential bias by estimating missing studies and incorporating hypothetical datasets into primary analysis to derive adjusted pooled estimates (25). All statistical analyses were performed using the *meta* package (R 4.1.3) with statistically significance at *P* < 0.05.

## Results

### Study selection

**Figure 1:**
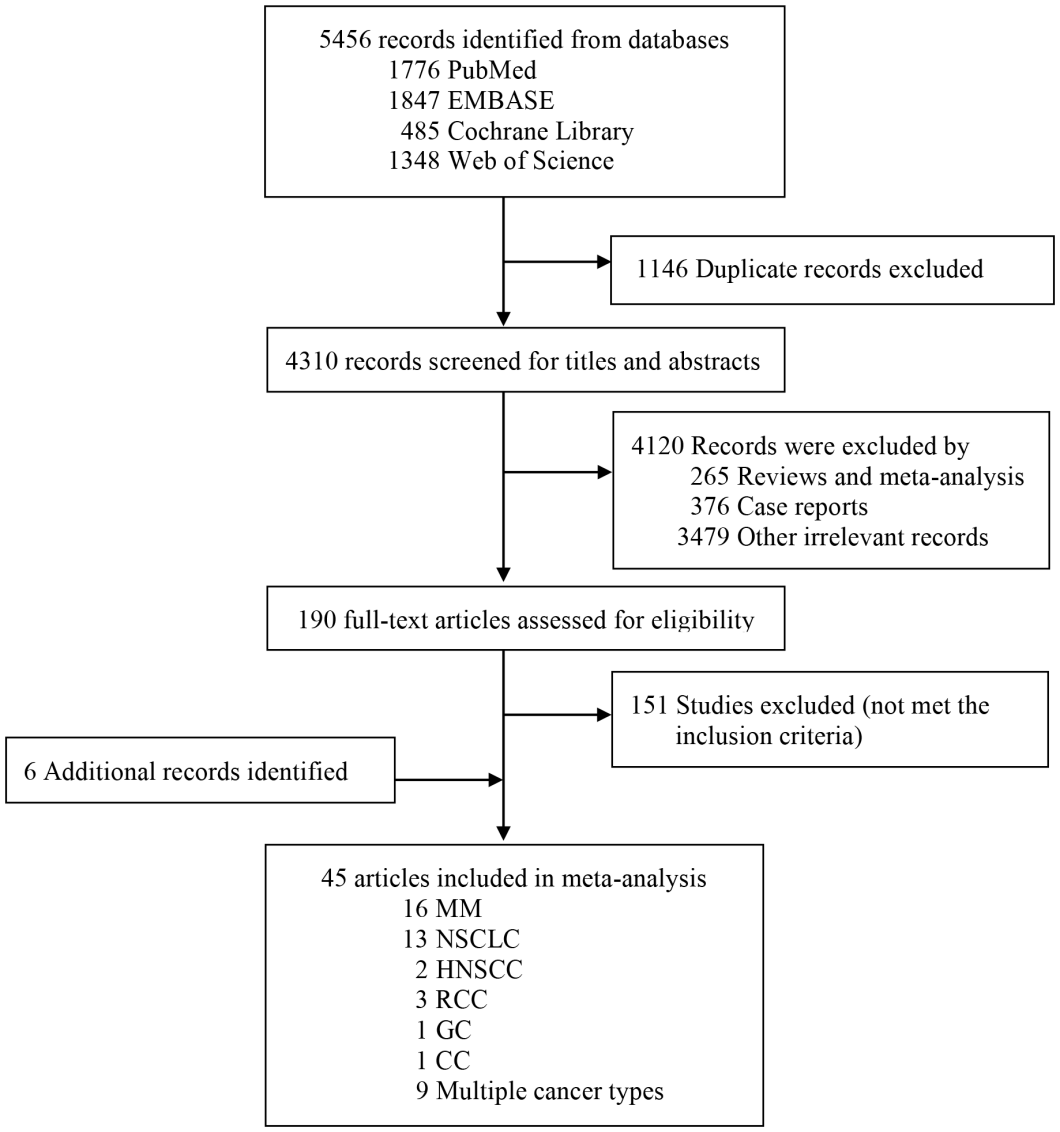
Flowchart of study selection for meta-analysis.

Our systematic search yielded 5,456 records from databases, **Figure 1** details the study selection process. After screening titles and abstracts, we identified 190 potentially eligible articles examining associations between cirAEs and prognosis in patients receiving ICIs therapy. Detailed full-text assessment excluded 151 articles failing to meet the inclusion criteria. The final meta-analysis included 45 articles involving 26,817 patients (10, 13–15, 18, 20, 22, 26–63).

### Study characteristics

The main characteristics of the 45 included studies were summarized in **Supplementary Table S1**. The majority were retrospective studies (10, 13–15, 18, 20, 22, 26–28, 30, 32–35, 37–56, 58, 59, 61–63), with 2 prospective studies (29, 31) and 3 clinical trials (36, 57, 60). By tumor type, 16 studies focused on melanoma (26, 30, 31, 35, 36, 38, 46–48, 50, 56, 57, 59, 60, 63), 13 on NSCLC (27–29, 32–34, 40, 41, 45, 49, 54, 58, 61), 2 in HNSCC (37, 53), 3 in RCC (10, 15, 22), 1 in GC (62) and 1 in CC (51), with 9 studies involving mixed tumor types. Nearly half of the studies were conducted in Asia-Pacific countries(n=21) (10, 18, 20, 27, 28, 31, 34, 40–42, 45–48, 53, 56, 59–63), 11 in European countries (15, 29, 30, 32, 33, 35–37, 43, 50, 54), 11 in USA (13, 14, 22, 26, 38, 39, 44, 52, 55, 57, 58) and two involved global populations (49, 51). Most studies (n=29) reported both OS and PFS as clinical outcomes (10, 20, 22, 26–30, 32–35, 40, 43–45, 47, 49–54, 57–59, 61–63), while 9 studies provided only OS data (13, 14, 18, 37–39, 46, 56) and 7 provided only PFS data (15, 31, 36, 41, 42, 55, 60). Regarding ICIs type, most studies investigated PD-1 and/or PD-L1 inhibitors (13, 20, 22, 27–38, 40–42, 44–50, 52, 54–56, 58–63), and 10 studies examined CTLA-4 inhibitors (10, 14, 15, 18, 26, 39, 43, 51, 53, 57). The NOS quality scores of eligible studies in the meta-analysis ranged from 5 to 9 stars (mean ± SD,7.16 ± 1.17), with the median follow-up durations ranged from 3.8 to 54.2 months (**Supplementary Table S1**).

### Association of cirAEs occurrence with OS and PFS

**Figure 2:**
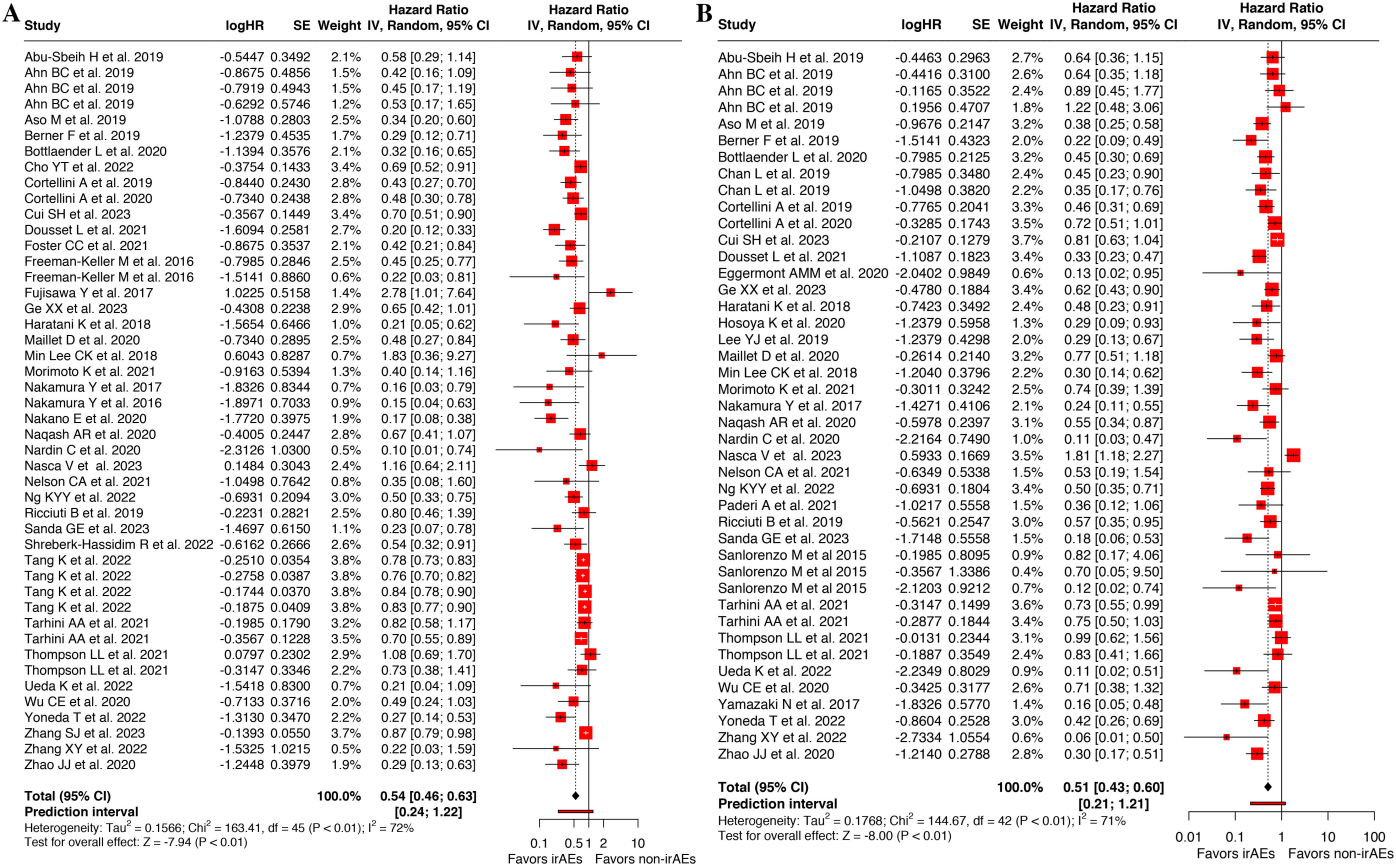
Forest plot of the association between cutaneous immune-related adverse events (cirAEs) and overall survival **(A)** and progression-free survival **(B)**.

Among 37 articles examining OS outcomes (10, 13, 14, 18, 20, 22, 26–30, 32–35, 37–40, 43–54, 56–59, 61–63), 14 studies showed no association between the incidence of cirAEs and OS in patients. Pooled analysis of 46 OS datasets (10, 13, 14, 18, 20, 22, 26–30, 32–35, 37–40, 43–54, 56–59, 61–63) demonstrated that the cirAEs following ICI therapy were significantly associated with prolonged OS in patients(HR, 0.54 [95%CI, 0.46-0.63]; *I^2^* = 72.5%; P < 0.001), as shown in **Figure 2A**. Analysis of PFS across 36 studies (10, 15, 20, 22, 26–36, 40–45, 47, 49–55, 57–63) indicated that 12 showed no difference in PFS between patients with cirAEs and non-cirAEs. Pooled analysis of 43 PFS datasets (10, 15, 20, 22, 26–36, 40–45, 47, 49–55, 57–63) confirmed superior PFS outcomes in patients with cirAEs receiving ICI therapy compared with those without cirAEs (HR, 0.51 [95%CI, 0.43-0.60]; *I^2^* = 71.0%; P < 0.001), as illustrated in **Figure 2B**.

**Figure 3:**
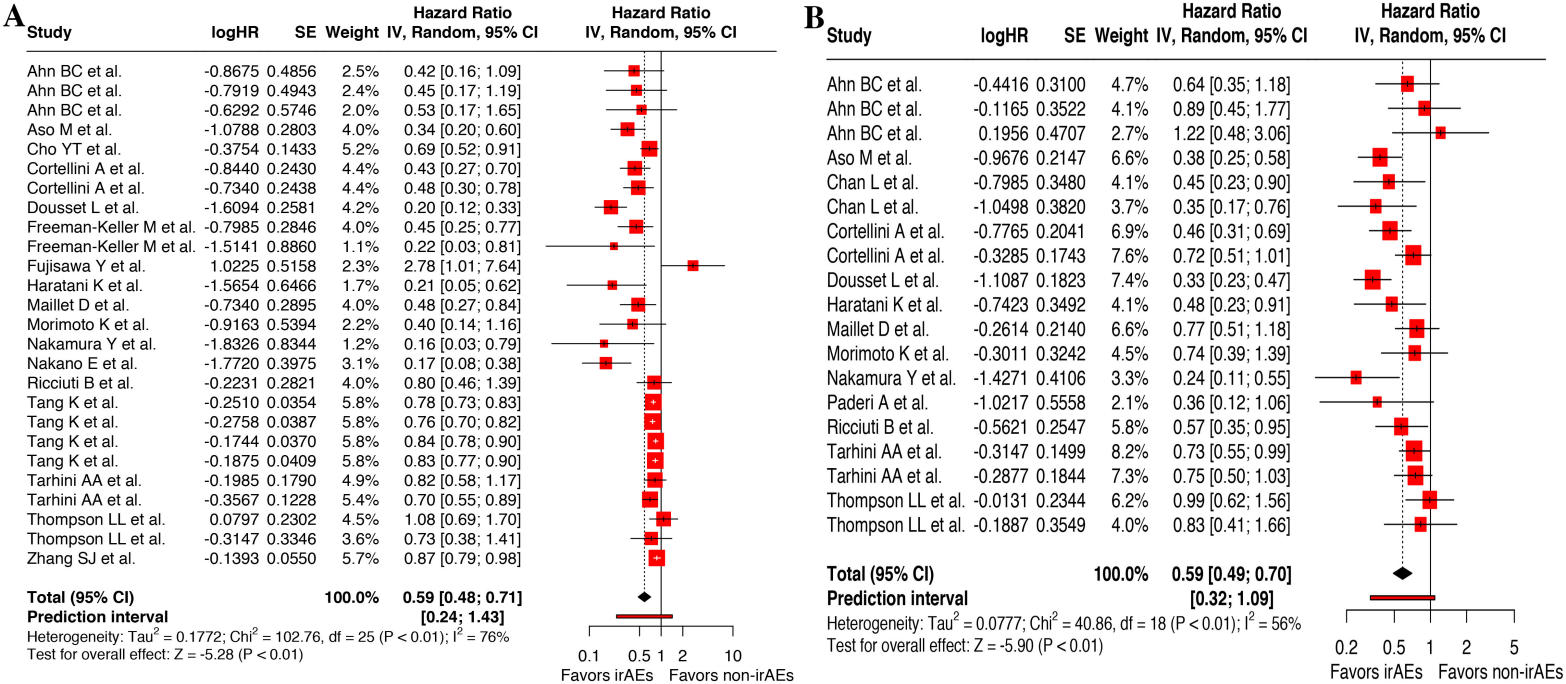
Landmark analysis of the association between cutaneous immune-related adverse events (cirAEs) and overall survival **(A)** and progression-free survival **(B)**.

Besides, landmark analysis was adopted to reduce guarantee-time bias, which incorporated 26 available records for OS (13, 14, 18, 27, 28, 32, 33, 35, 38–40, 43, 45, 47, 48, 54, 57, 58) and 19 for PFS (15, 27, 28, 31–33, 35, 40, 43, 45, 47, 54, 57, 58). Meta-analysis of these corresponding data demonstrated a significant improved OS (HR, 0.59 [95%CI, 0.48-0.71]; *I^2^* = 75.7%; *P* < 0.001) (**Figure 3A**) and PFS (HR, 0.59 [95%CI, 0.49-0.69]; *I^2^* = 56.0%; *P* < 0.001) in patients who experienced cirAEs compared with those who did not (**Figure 3B**).

### Subgroup analysis

#### Stratification by geographic region

The associations between the presence of cirAEs and improved OS as well as PFS were consistently observed among patients in every region, including Asia-Pacific, Europe and North American, with moderate heterogeneity (**Table 1**).

**Table 1:** Subgroup analyses of survival prognosis based on methods and study characteristics.

Characteristic	Overall survival				Progression-free survival			
	Data	Hazard ratio (95%CI)	*I^2^(%)*	P value	Data	Hazard ratio (95%CI)	*I^2^(%)*	P value
Sum	46	0.54 (0.46-0.63)	72.5	<0.001	43	0.51 (0.43-0.60)	71.0	<0.001
Landmark analysis	26	0.59 (0.48-0.71)	75.7	<0.001	19	0.59 (0.49-0.69)	56.0	<0.001
Geographic region								
Asia-Pacific	20	0.44 (0.34-0.57)	57.8	<0.001	20	0.49 (0.39-0.59)	59.8	<0.001
Europe	8	0.39 (0.27-0.55)	58.9	<0.001	10	0.45 (0.34-0.59)	62.9	<0.001
North America	15	0.80 (0.77-0.83)	48.2	<0.001	11	0.68 (0.58-0.82)	43.0	<0.001
Global	2	0.83 (0.57-1.21)	49.4	0.33	2	1.01 (0.31-3.24)	94.0	0.09
Study design								
Retrospective	43	0.53 (0.45-0.62)	73.7	<0.001	36	0.53 (0.44-0.63)	72.2	<0.001
Prospective	–	–	–	–	3	0.34 (0.22-0.53)	0.0	<0.001
Trial	2	0.74 (0.60-0.89)	0.0	0.002	4	0.43 (0.19-0.99)	68.8	0.04
Sample size								
≥ 100	34	0.58 (0.49-0.67)	73.8	<0.001	27	0.59 (0.49-0.70)	73.8	<0.001
< 100	12	0.42 (0.32-0.56)	49.2	<0.001	16	0.34 (0.25-0.46)	38.3	<0.001
Multi-centers								
S	16	0.76 (0.70-0.82)	63.9	<0.001	14	0.52 (0.38-0.73)	80.9	<0.001
M	30	0.46 (0.37-0.57)	61.6	<0.001	29	0.48 (0.30-0.57)	61.0	<0.001
Analysis								
UVA	14	0.78 (0.74-0.82)	58.4	<0.001	17	0.45 (0.35-0.59)	59.9	<0.001
MVA	32	0.49 (0.40-0.60)	74.4	<0.001	26	0.55 (0.44-0.67)	76.1	<0.001
cirAEs type								
Mixed	33	0.63 (0.55-0.72)	65.5	<0.001	29	0.54 (0.44-0.66)	73.4	<0.001
Bullous pemphigoid	3	0.70 (0.41-1.22)	0.0	0.21	–	–	–	–
Drug hypersensitivity or eruption	3	0.74 (0.63-0.87)	26.3	<0.001	–	–	–	–
Eczematous	2	0.69 (0.50-0.94)	0.0	<0.001	–	–	–	–
Lichenoid or lichen planus-like lesion	2	0.51 (0.38-0.69)	0.0	<0.001	–	–	–	–
Maculopapular	2	0.83 (0.68-1.03)	0.0	0.10	–	–	–	–
Mucositis	4	1.19 (0.95-1.49)	47.4	0.12	–	–	–	–
Pruritus	3	0.70 (0.62-0.79)	0.0	<0.001	–	–	–	–
Psoriasis	2	0.63 (0.48-0.83)	6.3	0.001	–	–	–	–
Rash	9	0.69 (0.64-0.74)	23.0	<0.001	7	0.69 (0.58-0.82)	0.0	<0.001
Vitiligo	9	0.23 (0.18-0.33)	9.7	<0.001	6	0.28 (0.21-0.38)	0.0	<0.001
Cancer type								
GC	2	0.62 (0.20-1.92)	42.9	0.41	–	–	–	–
HNSCC	4	0.64 (0.42-0.99)	60.1	0.04	–	–	–	–
MM	15	0.40 (0.28-0.59)	73.3	<0.001	13	0.43 (0.32-0.56)	66.8	<0.001
NSCLC	15	0.57 (0.49-0.66)	49.7	<0.001	16	0.63 (0.56-0.71)	51.6	<0.001
RCC	2	0.22 (0.08-0.59)	0.0	0.002	3	0.22 (0.11-0.43)	0.0	<0.001
Mixed	10	0.80 (0.78-0.83)	38.1	<0.001	8	0.57 (0.45-0.72)	34.0	<0.001
ICI type								
Anti-PD(L)-1	36	0.48 (0.40-0.58)	75.1	<0.001	34	0.48 (0.41-0.57)	58.9	<0.001
Mixed	8	0.73 (0.54-0.98)	68.4	0.02	6	0.62 (0.33-1.14)	87.3	0.10
ICI-Combination	13	0.74 (0.59-0.91)	58.8	0.004	10	0.52 (0.30-0.88)	83.6	<0.001
ICI-Monotherapy	33	0.49 (0.41-0.58)	76.2	<0.001	32	0.48 (0.41-0.57)	58.4	<0.001

ICIs, immune checkpoint inhibitor; MM, melanoma; NSCLC, non–small cell lung cancer; HNSCC, head and neck squamous cell carcinoma; RCC, renal cell carcinoma; GC, gastric cancer; S, single center; M, multi-centers; UVA, univariate analysis; MVA, multivariate analysis; PD-1, programmed cell death 1; PD-L1, programmed cell death ligand 1; CTLA-4, cytotoxic T lymphocyte antigen 4.

#### Stratification by study quality

As the eligible articles were classified as retrospective, prospective and clinical trials, the positive association between cirAE occurrence and improved OS remained significant in both clinical trials (HR, 0.74 [95%CI, 0.60-0.89]; *I^2^* = 0.0%; *P* < 0.001) and retrospective studies(HR, 0.53 [95%CI, 0.45-0.62]; *I^2^* = 73.7%; *P* < 0.001), while this association was consistently observed across all three study designs for PFS (**Table 1**).

Similarly, subgroup analyses based on sample size, analysis model and multi-centers demonstrated that the cirAEs occurrence would significant prolonged patient survival and reduced risk of progression, which still remains consistent findings although subgroups stratified by study quality characteristics yielded inconsistent results (**Table 1**).

#### Stratification by cirAEs subtype

In the subgroup analysis based on cirAEs subtype, favorable survival was observed in patients with vitiligo, with the most pronounced OS (HR, 0.23 [95%CI, 0.18-0.33]; *I^2^* = 9.7%; *P* < 0.001) and PFS (HR, 0.28 [95%CI, 0.21-0.38]; *I^2^* = 0.0%; *P* < 0.001). Similarly, the prolonged OS (HR, 0.69 [95%CI, 0.64-0.74]; *I^2^* = 23.0%; *P* < 0.001) and PFS (HR, 0.69 [95%CI, 0.58-0.82]; *I^2^* = 9.7%; *P* < 0.001) in patients with rash. Significant benefit in OS were also observed in (HR, 0.74 [95%CI, 0.63-0.87]; *I^2^* = 26.3%; *P* < 0.001), eczematous (HR, 0.69 [95%CI, 0.50-0.94]; *I^2^* = 0.0%; *P* < 0.001), lichenoid or lichen planus-like lesion (HR, 0.51 [95%CI, 0.38-0.69]; *I^2^* = 0.0%; *P* < 0.001), pruritus (HR, 0.70 [95%CI, 0.62-0.79]; *I^2^* = 0.0%; *P* < 0.001), psoriasis (HR, 0.63 [95%CI, 0.48-0.83]; *I^2^* = 6.3%; *P* = 0.001), but not in bullous pemphigoid, maculopapular and mucositis (*P*>0.05) (**Table 1**).

#### Stratification by cancer type

Subgroup analyses by cancer type indicated that Favorable survival was observed in patients with RCC, NSCLC and MM, with the most pronounced OS (HR, 0.22 [95%CI, 0.08-0.59]; *I^2^* = 0.0%; *P* = 0.002) and PFS (HR, 0.22 [95%CI, 0.11-0.43]; *I^2^* = 0.0%; *P* < 0.001) for RCC; and only OS benefit in HNSCC (HR, 0.64 [95%CI, 0.42-0.99]; *I^2^* = 60.1%; *P* = 0.04), while no substantial relationship in GC (**Table 1**).

#### Stratification by ICIs type

Among ICIs chosen in the eligible studies, anti-PD-(L)1 inhibitor predominated. The presence of cirAEs in patients with anti-PD-(L)1 therapy was significantly associated with improved (HR, 0.48 [95%CI, 0.40-0.58]; *I^2^* = 75.1%; *P* < 0.001) and PFS (HR, 0.48 [95%CI, 0.41-0.57]; *I^2^* = 58.9%; *P* < 0.001). Similarly, patients with cirAEs who received ICIs-monotherapy or ICIs-combination treatment demonstrated prolonged OS and PFS (**Table 1**).

#### Sensitivity analysis and publication bias

Sensitivity analyses were performed by sequentially excluding individual studies. The significant associations of ICI-related cirAEs incidence with OS and PFS remained robust across all iterations, with the overall estimates showing minimal variation (**Supplementary Figure S1**). Furthermore, sensitivity analysis restricted to studies included for landmark analysis supported the robustness of the findings (**Supplementary Figure S2**).

Publication bias were assessed by Begg’s test along with the funnel plot and Egger’s regression test. In terms of OS and PFS data, the asymmetry funnel plots suggested potential publication bias (**Supplementary Figures S3**, **S4**), which was validated by Begg’s test and Egger’s test (*P* < 0.05). Subsequently, the trim-and-fill method were used to quantified the impact of publication bias. After imputing 18 missing data for OS and 13 for PFS, the adjusted pooled estimates remained consistent with primary findings (OS: HR, 0.74 [95%CI, 0.60-0.90]; *I^2^* = 77.4%; *P* = 0.003; PFS: HR, 0.63 [95%CI, 0.52-0.76]; *I^2^* = 74.1%; *P* = 0.04), which supported the robustness and stability of the findings.

## Discussion

This meta-analysis substantiated that cirAEs could serve as clinical biomarkers predicting improved survival outcomes across diverse malignancies among patients receiving ICI therapy. Although the treatment innovation led by ICIs has reshaped the paradigm of tumor treatment, the paradoxical relationship between immunotoxicity and its efficacy has not been clarified. Our findings—derived from 45 studies encompassing 26,817 patients—provide the most comprehensive evidence to date that cirAE incidence indicates significant OS and PFS advantages, which are consistent with previous meta-analysis (6). To address the potential association while minimizing potential bias related to the varying duration of ICI exposure, landmark analyses were performed in patients with complete survival data spanning at least 6 weeks. The results further supported the hypothesis that cirAEs were significantly associated with long-term clinical benefits in cancer patients subjected to ICI treatment, which may be attributed to the bystander effect of reactivated T cells (4).

Our results of subgroup analyses demonstrated the differential prognostic impact of cirAE in specific populations. Firstly, regarding cirAE subtypes, the survival benefit associated with vitiligo was the most favorable for both OS and PFS, which was not only consistent with previous meta-analysis (6), but also aligned with studies among patients with NSCLC and MM (34–36, 38, 46–48, 50, 60). Besides, favorable survival were also observed in patients with vitiligo and drug hypersensitivity or eruption, which were different with previous meta-analysis (6). Significant OS advantages also emerged for eczematous, lichenoid or lichen planus-like lesion, pruritus and psoriasis, but not for bullous pemphigoid, maculopapular or mucositis. However, the exact mechanism of cirAEs and the discrepancy between ICIs type has not been fully clarified. Besides, previous studies have suggested that epitope spreading may contribute to robust antitumor activity through clonal diversification of T-cell responses. This mechanism may help explain the superior survival outcomes observed among MM patients with vitiligo compared to those without vitiligo. Meanwhile, the tissue homing theory, which proposes that circulating lymphocytes migrate to sites of initial antigen encounter through receptors on reactivated systemic memory T cells, could similarly explain the pathogenesis of certain cirAEs (14, 64). Furthermore, rash induced by PD-1 blockade might be explained by disrupted regulatory T-cell(Treg)-mediate immunosuppression via PD-1 pathway modulation, as demonstrated in murine graft-versus-host-disease (GVHD) models. PD-1 inhibition might be related to typical cutaneous changes of GVHD and CD8+ T cell expansion (37). The resultant dysregulated immune activation from checkpoint inhibitors targeting these pathways brings a spectrum of irAEs including cirAEs. This pathophysiological framework provides a biologically plausible basis for the association of cirAE with improved prognosis, where heightened autoimmune toxicity may serve as a surrogate marker for enhanced antitumor immune surveillance (4, 6, 65).

Secondly, regarding tumor types, the associations between cirAEs and both OS and PFS were pronounced in patients with NSCLC, MM, HNSCC and RCC, but not in those with GC. Expected to NSCLC and MM consisting with previous results (6), we also confirmed that the association existed in RCC and HNSCC patients, which RCC patients might have a marginally larger benefit among different cancer types. As the significant improvement in prognosis demonstrated by numerous clinical trials that ICIs have been adopted in various tumor types and have altered the therapeutic landscape among cancer patients (5, 10). Thirdly, geographic variations were observed, with Asia-Pacific and European cohorts showed marginally greater OS benefits than North American patients. This may potentially reflect gene-environment interactions involving both nonmodifiable factors such as genetic susceptibility and modifiable risk factors including daily behavior, diet and physical activity. Nevertheless, the underlying biological mechanisms regarding disparities of treatment efficacy remain unclear (4, 64). Fourth, differential survival impacts were also observed across ICIs as anti-PD-(L)1 inhibitors conferred stronger survival advantage, however the association between cirAEs and survival for patients receiving anti-CTLA-4 monotherapy could not be subjected to a formal meta-analysis due to a lack of available studies, only two OS datasets and one PFS data from a single study was included. Given the evidence base remains far too limited to draw any meaningful conclusions, further investigation is expected. The survival benefit discrepancy between ICI types observed in this study has been reported in previous research, suggesting fundamental differences in immune checkpoint biology. CTLA-4 modulates early T-cell activation in lymphoid tissues through attenuation of co-stimulatory signaling, while PD-1 primarily regulates late-stage T-cell exhaustion in peripheral tissues by suppressing effector functions (11, 66, 67). This divergence may explain why cirAEs exhibit superior predictive value for PD-(L)1 inhibitor efficacy (18). Additionally, this meta-analysis incorporated all currently available studies, including retrospective, prospective and clinical trials. Intriguingly, definite benefits of OS and PFS in cirAEs group were observed in pooled analyses of retrospective studies and clinical trials, and benefits of PFS in prospective studies but failed of OS. Of note, retrospective studies are virtually vulnerable to several forms of appraisal bias. Due to the limited available data, well-designed large prospective cohort studies with confounding control are needed to generate further insight.

To our knowledge, this study represents the most comprehensive systematic review and the most updated meta-analysis using all currently available data for examining the association between cirAEs incidence and the survival benefits of ICIs treatment. Subgroup analyses, sensitivity analyses, and landmark analyses were conducted to minimize potential confounding effects, supporting the robustness of the findings. Nevertheless, certain limitations of this meta-analysis should be acknowledged. First, while incorporating studies with diverse designs enhances generalizability, the predominance of retrospective data may introduce inherent methodological risks of recall bias and selection bias. Still, the consistent observation of significant positive associations between cirAE incidence and survival outcomes across diverse study designs strengthen the robustness. Second, the substantial heterogeneity observed in the primary meta-analyses necessitates cautious interpretation. Heterogeneity could be attributed to methodological differences in study design, therapeutic regimens (anti-CTLA-4, anti-PD-1/PD-L1, or combination therapy), and tumor microenvironment characteristics across tumor types. Consequently, the pooled hazard ratio should be viewed as a summary measure that may not be uniformly applicable to all clinical scenarios, and our findings are best interpreted as generating hypotheses for future research in more defined patient subgroups. Third, detected publication bias in both OS and PFS analyses might exaggerate the pooled effect estimates, though the result from trim-and-fill analysis confirmed the stability after imputation of missing studies. Fourth, the limited data source of specific subgroups, particularly rare cirAE subtypes and understudied tumor types, restrict a universal conclusion. The findings from our subgroup analyses should be viewed as hypothesis-generating due to the issue of multiple testing and the lack of statistical correction. Future large-scale prospective cohort studies are needed to generate further insight and elucidate their underlying mechanisms. Fifth, this meta-analysis incorporated retrospective, prospective and clinical trials. Across study designs, retrospective and clinical trial data consistently demonstrated OS and PFS benefits, while prospective studies showed only PFS improvement. It should be noted that retrospective studies (accounting for 78% of included studies) are vulnerable to several forms of appraisal bias and might introduce the risk of immortal time bias, as the development of a cirAE is a time-dependent event that occurs after treatment initiation. Although we performed a landmark analysis to mitigate this bias, which may not fully account for all residual confounding. The findings for specific, rare cirAE subtypes (e.g., bullous pemphigoid, mucositis) and for less common cancer types (e.g., gastric cancer) are fundamentally limited by a lack of statistical power. The non-significant results for these subgroups should not be interpreted as evidence of no association; rather, they are inconclusive and highlight an area where future, future large-scale prospective cohorts with confounding control are warranted to corroborate these associations.

## Conclusions

This comprehensive meta-analysis of 45 studies indicated that cirAEs occurrence was associated with significantly prolonged survival among tumor patients receiving ICIs therapy, suggesting cirAEs may serve as potential prognostic biomarkers. The association was particularly pronounced in MM, RCC and NSCLC patients and specific cirAE subtypes like vitiligo and rash. These findings should be interpreted cautiously and warrant validation through large-scale prospective studies.

## Data Availability

The original contributions presented in the study are included in the article/**Supplementary Material**. Further inquiries can be directed to the corresponding author.
