# Supplementary material for: Cutaneous adverse events associated with the efficacy and benefit of immune checkpoint inhibitors: a systematic review and meta-analysis
Source: Front Oncol. 2025 Nov 7;15:1689519. doi: 10.3389/fonc.2025.1689519 (PMC12634405; doi:10.3389/fonc.2025.1689519)
Supplement: Supplementary file 1 [file DataSheet1.docx]

**Table S1: Characteristics of Included Studies**

| Author | Design | Region | Centers | Cancer | Follow time (months) | ICI types | Patients  (M/F) | Median age (y) | cirAEs | cirAEs type | OS  (HR,95%CI) | PFS  (HR,95%CI) | Analysis /Landmark | | NOS |
| --- | --- | --- | --- | --- | --- | --- | --- | --- | --- | --- | --- | --- | --- | --- | --- |
| Abu-Sbeih H et al (1) | RC | USA | S | MM | 20.4 | all/Comb- | 346(222/124) | 58.4±15.7 | 30 | any | 0.58(0.29-1.14) | 0.64(0.36-1.15) | MVA/- | 8 | |
| Ahn BC et al (2) | RC | Korea | S | NSCLC | 17.0 | PD-1/Mono- | 111(113/42) | 64(35-85) | 30 | any | 6-w:0.42(0.162-1.087);  12-w:0.453(0.172-1.194);  24-w:0.533(0.173-1.645) | 6-w:0.643(0.35-1.18);  12-w:0.89(0.446-1.774);  24-w:1.216(0.483-3.057) | MUA/6-w, 12-w, 24-w | 6 | |
| Aso M et al (3) | RC | Japan | S | NSCLC | - | PD-1/Mono- | 155(117/38) | 68(31-88) | 51 | any | 0.34(0.2-0.6) | 0.38(0.25-0.58) | MVA/6-w | 6 | |
| Berner F et al (4) | PC | Switzerlan | M | NSCLC | - | PD-1/Mono- | 73(44/29) | 68.1±8.9 | 25 | any | 0.29(0.12-0.71) | 0.22(0.09-0.49) | UVA/- | 8 | |
| Bottlaender L et al (5) | RC | France | S | MM | 4.1 | PD-L1/Mono- | 189(109/80) | 64.6(50–74.7) | 39 | any | 0.32 (0.16, 0.65) | 0.45(0.30, 0.69) | UVA/- | 7 | |
| Chan L et al (6) | PC | Australia | S | MM | 40.7 | PD-L1/Mono- | 82(51/31) | 59(19-82) | 33 | any | - | 6-m:0.45(0.23-0.9);  12-m:0.35(0.17-0.76) | UVA/6-m, 12-m | 8 | |
| Cho YT et al (7) | RC | Taiwan, China | S | Mixed | 6.8 | all/Comb- | 468(306/162) | 59.6±13.3 | 128 | any | 0.687(0.519-0.91) | - | MVA/L | 7 | |
| Cortellini A et al (8) | RC | Italy | M | NSCLC | 11.2 | PD-1/Mono- | 559(379/180) | 69(24-88) | 59 | any | 0.43(0.27-0.70) | 0.46(0.31-0.69) | MVA/6-w | 8 | |
| Cortellini A et al (9) | RC | Italy | M | NSCLC | 14.8 | PD-1/Mono- | 877(573/304) | 70.3(28-92) | 100 | any | 0.48(0.3-0.78) | 0.72(0.51-1.01) | MVA/6-w | 9 | |
| Cui SH et al (10) | RC | China | S | NSCLC | 14.8 | PD-(L)1/Mono- | 135(108/27) | 66(37-83) | 44 | vitiligo | 0.70 (0.51-0.90) | 0.81 (0.63-1.04) | UVA/- | 7 | |
| Dousset L et al (11) | RC | France | S | MM | 27.5 | PD-1/Mono- | 457(262/195) | 66(24-94) | 85 | vitiligo | 0.20 (0.12-0.33) | 0.33 (0.23-0.47) | MVA/12-w | 6 | |
| Eggermont AMM et al (12) | TR | Belgium | M | MM | - | PD-1/Mono- | 89(67/22) | 65(36–92) | 18 | vitiligo | - | 0.13 (0.02-0.95) | MVA/- | 9 | |
| Foster CC et al (13) | RC | France | S | HNSCC | 8.4 | PD-(L)1/Mono- | 108(82/26) | 60 (25-85) | 21 | any | 0.42(0.21-0.84) | - | MVA/- | 8 | |
| Freeman-Keller M et al (14) | RC | USA | S | MM | 35 | PD-1/Mono- | 148(87/64) | 17-90 | 64 | rash  vitiligo | rash:0.45(0.251-0.766);  vitiligo:0.22(0.025-0.806) | - | MVA/12-w | 8 | |
| Fujisawa Y et al (15) | RC | USA | S | MM | - | all/Comb- | 60(30/30) | 63.3(31-85) | 9 | any | 2.78(1.012-7.642) | - | MVA/2-m | 6 | |
| Ge XX et al (16) | RC | China | S | Mixed | 15.2 | PD-(L)1/Comb- | 241(180/61) | 60.9 (14–86) | 88 | any | 0.65 (0.42-1.01) | 0.62 (0.43_0.90) | MVA/- | 7 | |
| Haratani K et al (17) | RC | Japan | S | NSCLC | - | PD-1/Mono- | 134(90/44) | 68(33-85) | 43 | rash | 0.209(0.049-0.618) | 0.476(0.232-0.912) | MVA/6-w | 7 | |
| Hosoya K et al (18) | RC | Japan | M | NSCLC | - | PD-1/Mono- | 148(124/20) | 71(39-87) | 25 | rash | - | 0.29(0.09-0.93) | UVA/- | 6 | |
| Lee YJ et al (19) | RC | Korea | S | Mixed | - | PD-1/Mono- | 211(37/86) | 61(21-91) | 35 | any | - | 0.291(0.125-0.674) | UVA/- | 7 | |
| Maillet D et al (20) | RC | France | S | Mixed | - | all/Mono- | 435(295/140) | 66(58-73) | 30 | rash | 0.48(0.27-0.84) | 0.77(0.51-1.18) | MVA/L | 8 | |
| Min Lee CK et al (21) | RC | USA | S | Mixed | - | PD-1/Mono- | 114(62/52) | 65.9±12.4 | 20 | dermatitis | 1.83 (0.36, 9.27) | 0.30 (0.14, 0.62) | UVA/- | 6 | |
| Morimoto K et al (22) | RC | Japan | M | NSCLC | 14.8 | PD-(L)1/Comb- | 70(51/19) | 69.5(43-85) | 20 | rash | 0.40 (0.14-1.16) | 0.74 (0.39-1.39) | MVA/12-w | 8 | |
| Nakamura Y et al (23) | RC | Japan | S | MM | - | PD-1/Mono- | 98(52/46) | 66.5(17-93) | 13 | vitiligo | 0.15(0.04-0.63) | - | UVA/- | 6 | |
| Nakamura Y et al (24) | RC | Japan | S | MM | - | PD-1/Mono- | 35(18/17) | 67.8(40-85) | 9 | vitiligo | 0.16(0.03-0.79) | 0.24(0.11-0.55) | UVA/ L | 6 | |
| Nakano E et al (25) | RC | Japan | S | MM | 21.5 | PD-1/Mono- | 128(70/58) | 66(17–92) | 30 | vitiligo | 0.17 (0.08-0.38) | - | MVA/20-w | 7 | |
| Naqash AR et al (26) | RC | Global | M | NSCLC | - | PD-1/Mono- | 531(307/224) | 65(28-93) | 33 | any | 0.67(0.41-1.07) | 0.55(0.34_0.87) | MVA/- | 5 | |
| Nardin C et al (27) | RC | France | S | MM | 14.6 | PD-1/Comb- | 111(58/53) | 67(25-89) | 32 | vitiligo | 0.099(0.013-0.737) | 0.109(0.025-0.471) | MVA/- | 5 | |
| Nasca V et al (28) | RC | Global | M | CC | - | all/Comb- | 331(180/151) | 60(47-70) | 44 | any | 1.16 (0.64-2.11) | 1.81 (1.18-2.27) | MVA/- | 7 | |
| Nelson CA et al (29) | RC | USA | S | Mixed | - | PD-(L)1/Mono- | 71(56/15) | 73(50-93) | 25 | Bullous pemphigoid | 0.35 (0.08, 1.60) | 0.53 (0.19, 1.54) | UVA/- | 6 | |
| Ng KYY et al (30) | RC | Singapore | S | HNSCC | 25.1 | all/Comb- | 168(144/24) | 69(60-75) | 79 | any | 0.50 (0.33, 0.75) | 0.50 (0.35, 0.71) | MVA/- | 8 | |
| Paderi A et al (31) | RC | Italy | S | RCC | - | all/Mono- | 43(35/8) | 64(45-79) | 9 | any | - | 0.36 (0.12-1.06) | MVA/L | 7 | |
| Ricciuti B et al (32) | RC | Italy | M | NSCLC | 26 | PD-1/Mono- | 195(128/67) | 63(30-84) | 43 | rash | 0.8(0.46-1.39) | 0.57(0.35-0.95) | MVA/6-w | 9 | |
| Sanda GE et al (33) | RC | USA | S | RCC | 20.1 | PD-(L)1/Mono- | 70(49/21) | 69.5(28-91) | 9 | any | 0.23(0.07-0.78) | 0.18 (0.06-0.53) | MVA/- | 6 | |
| Sanlorenzo M et al (34) | RC | USA | S | Mixed | 3.75 | PD-1/Mono- | 83(52/31) | 66(18-90) | 35 | any | - | 10mg/kg-3w:0.82(0.17-4.06);  10mg/kg-2w:0.70(0.05-9.50);  2mg/kg-3w:0.12(0.02-0.74) | UVA/- | 7 | |
| Shreberk-Hassidim R et al (35) | RC | Israel | S | MM | 54.2 | PD-(L)1/Mono- | 95(63/32) | 60.4±14.4 | 34 | any | 0.54(0.32-0.91) | - | MVA/- | 7 | |
| Tang K et al (36) | RC | USA | M | Mixed | - | PD-(L)1/Mono- | 14016(7936/6080) | 68.2 | 7008 | any | 3-m:0.759(0.703-0.818);  6-m:0.778 (0.726-0.834);  9-m:0.84(0.781-0.903);  12-m:0.829(0.765-0.898) | - | UVA/3-m, 6-m, 9-m, 12-m | 8 | |
| Tarhini AA et al (37) | TR | USA | M | MM | - | CTLA-4 | 1034(670/364) | 54(18–80) | 496 | rash | 0.82(0.58-1.17);  grade2:0.70 (0.55-0.89) | 0.71(0.55-0.99);  grade2:0.75(0.5-1.03) | UVA/3-m | 8 | |
| Thompson LL et al (38) | RC | USA | S | NSCLC | - | PD-(L)1/Comb- | 227(110/117) | 66.8(58.8-76.3) | 50 | any | 1.083(0.69-1.701);  severe:0.73(0.379-1.407) | 0.987(0.624-1.564)  severe:0.828(0.413-1.66) | MVA/6-w | 5 | |
| Ueda K et al (39) | RC | Japan | S | RCC | - | all/Comb- | 35(31/4) | 66(42–80) | 17 | any | 0.214(0.042-1.087) | 0.107(0.022-0.512) | MVA/- | 6 | |
| Wu CE et al (40) | RC | Taiwan, China | S | MM | 9.1 | PD-1/Mono- | 49(22/27) | 61 | 19 | vitiligo | 0.49 (0.24-1.03) | 0.71(0.38-1.32) | UVA/- | 8 | |
| Yamazaki N et al (41) | TR | Japan | M | MM | 18.8 | PD-1/Mono- | 24(10/14) | 63(26–81) | 9 | vitiligo | - | 0.16(0.05-0.48) | UVA/- | 6 | |
| Yoneda T et al (42) | RC | Japan | M | NSCLC | 11.3 | PD-(L)1/Mono- | 435(344/91) | 69(30-90) | 46 | any | 0.269(0.136-0.53) | 0.423(0.258-0.695) | MVA/- | 9 | |
| Zhang SJ et al (43) | RC | USA | S | Mixed | 31.0 | all/Comb- | 3731(2036/1695) | 65.1 | 676 | any | 0.87 (0.79, 0.98);  MM:0.67(0.51-0.87);  HNSCC:0.81(0.53-1.23);  GC:0.85(0.61-1.17) | - | MVA/6-m | 9 | |
| Zhang XY et al (44) | RC | China | S | GC | - | PD-1/Comb- | 74(51/23) | >65:33,44.6% | - | any | 0.216(0.029-1.590) | 0.065 (0.008-0.501) | MVA/- | 8 | |
| Zhao JJ et al (45) | RC | China | S | MM | - | PD-1/Mono- | 93(54/39) | 52(22-78) | 40 | any | 0.288(0.132-0.628) | 0.297(0.172-0.513) | MVA/- | 7 | |

Abbreviations: ICIs, immune checkpoint inhibitor; MM, melanoma; NSCLC, non–small cell lung cancer; HNSCC, head and neck squamous cell carcinoma; RCC, renal cell carcinoma; GC, gastric cancer; CC, colorectal cancer; S, single center; M, multi-centers; UVA, univariate analysis; MVA, multivariate analysis; PD-1, programmed cell death 1; PD-L1, programmed cell death ligand 1; CTLA-4, cytotoxic T lymphocyte antigen 4; cirAEs, cutaneous immune-related adverse events; PFS, progression free survival; OS, overall survival; HR, hazard ratio; CI, confidence interval.


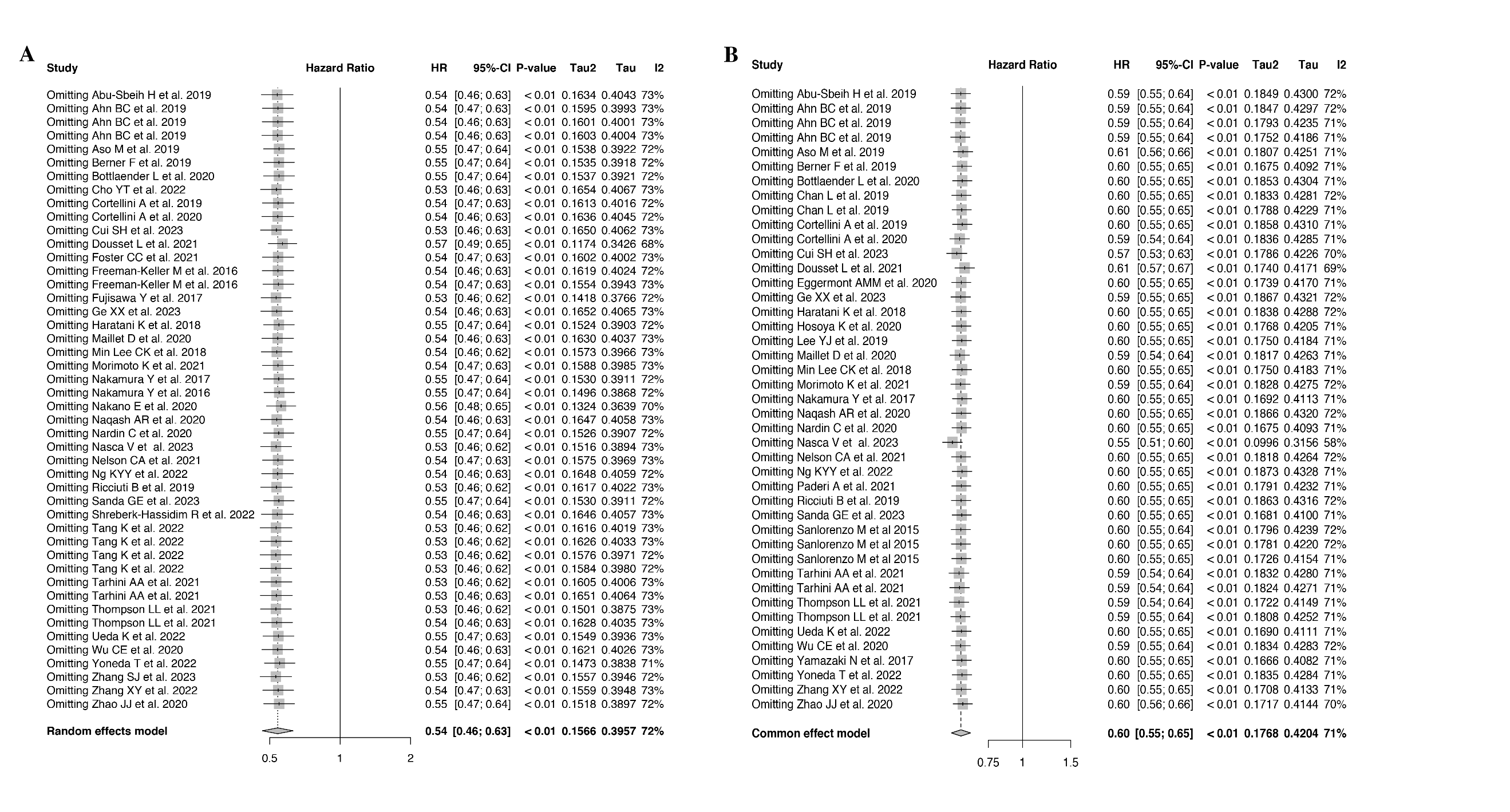
**Figure S1: Sensitivity analysis for Overall Survival (A) and Progression-Free Survival (B)**


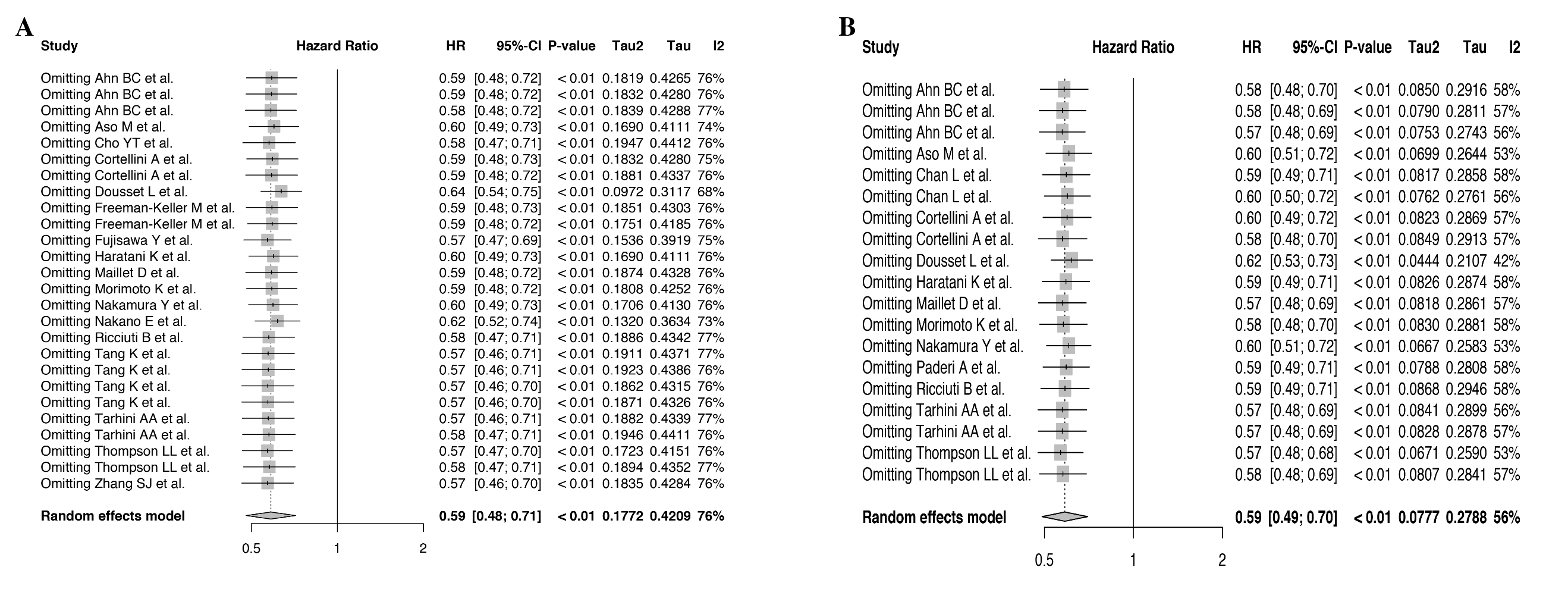


**Figure S2: Sensitivity analysis for Overall Survival (A) and Progression-Free Survival (B) in Studies with Landmark analysis**


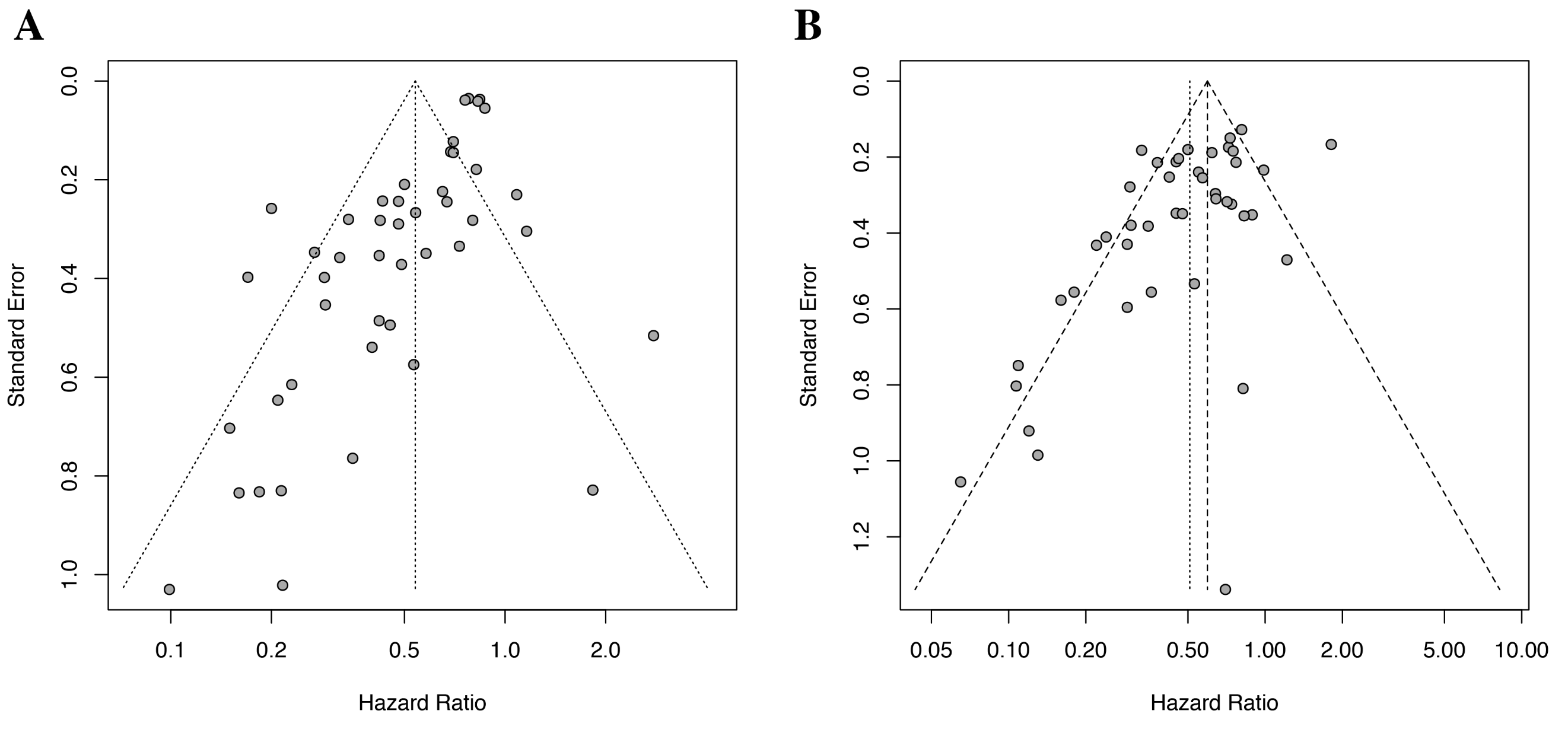


**Figure S3: Funnel plot for Overall Survival (A) and Progression-Free Survival (B) in Studies**


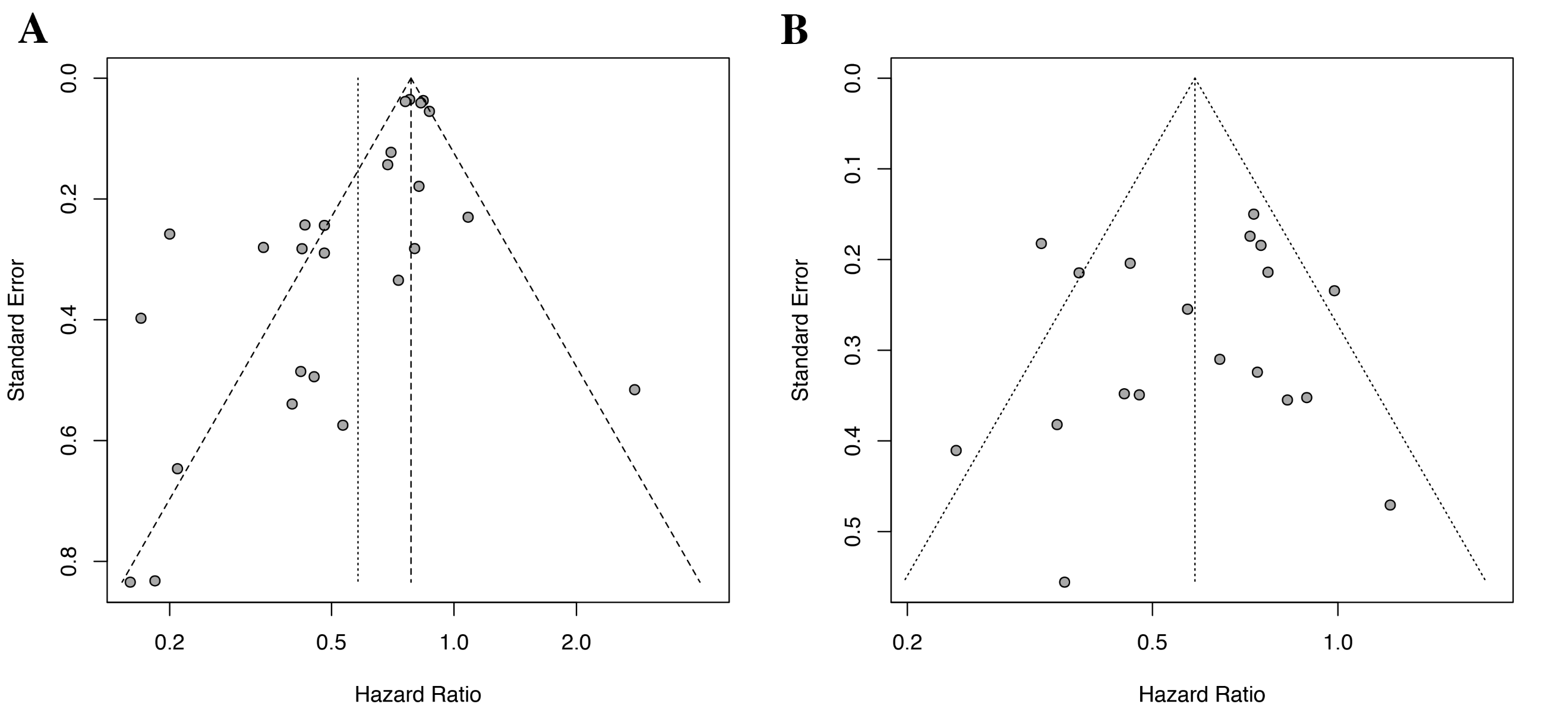


**Figure S4: Sensitivity analysis for Overall Survival (A) and Progression-Free Survival (B) in studies with landmark analysis**

**References**

1. Abu-Sbeih H, Ali FS, Qiao W, et al.: Immune checkpoint inhibitor-induced colitis as a predictor of survival in metastatic melanoma. Cancer Immunol Immunother 68: 553-561, 2019.

2. Ahn BC, Pyo KH, Xin CF, et al.: Comprehensive analysis of the characteristics and treatment outcomes of patients with non-small cell lung cancer treated with anti-PD-1 therapy in real-world practice. J Cancer Res Clin Oncol 145: 1613-1623, 2019.

3. Aso M, Toi Y, Sugisaka J, et al.: Association Between Skin Reaction and Clinical Benefit in Patients Treated with Anti-Programmed Cell Death 1 Monotherapy for Advanced Non-Small Cell Lung Cancer. Oncologist 25: e536-e544, 2020.

4. Berner F, Bomze D, Diem S, et al.: Association of Checkpoint Inhibitor-Induced Toxic Effects With Shared Cancer and Tissue Antigens in Non-Small Cell Lung Cancer. JAMA Oncol 5: 1043-1047, 2019.

5. Bottlaender L, Amini-Adle M, Maucort-Boulch D, Robinson P, Thomas L and Dalle S: Cutaneous adverse events: a predictor of tumour response under anti-PD-1 therapy for metastatic melanoma, a cohort analysis of 189 patients. J Eur Acad Dermatol Venereol 34: 2096-2105, 2020.

6. Chan L, Hwang SJE, Byth K, et al.: Survival and prognosis of individuals receiving programmed cell death 1 inhibitor with and without immunologic cutaneous adverse events. J Am Acad Dermatol 82: 311-316, 2020.

7. Cho YT, Lin YT, Yang CW and Chu CY: Cutaneous immune-related adverse events among Taiwanese cancer patients receiving immune checkpoint inhibitors link to a survival benefit. Sci Rep 12: 7021, 2022.

8. Cortellini A, Chiari R, Ricciuti B, et al.: Correlations Between the Immune-related Adverse Events Spectrum and Efficacy of Anti-PD1 Immunotherapy in NSCLC Patients. Clin Lung Cancer 20: 237-247.e231, 2019.

9. Cortellini A, Friedlaender A, Banna GL, et al.: Immune-related Adverse Events of Pembrolizumab in a Large Real-world Cohort of Patients With NSCLC With a PD-L1 Expression ≥ 50% and Their Relationship With Clinical Outcomes. Clin Lung Cancer 21: 498-508.e492, 2020.

10. Cui S, Ge X and Li X: [A Real-world Study on the Incidence and Outcome of Immune-related Adverse Events in Lung Cancer Patients]. Zhongguo Fei Ai Za Zhi 26: 257-264, 2023.

11. Dousset L, Pacaud A, Barnetche T, et al.: Analysis of tumor response and clinical factors associated with vitiligo in patients receiving anti-programmed cell death-1 therapies for melanoma: A cross-sectional study. JAAD Int 5: 112-120, 2021.

12. Eggermont AMM, Kicinski M, Blank CU, et al.: Association Between Immune-Related Adverse Events and Recurrence-Free Survival Among Patients With Stage III Melanoma Randomized to Receive Pembrolizumab or Placebo: A Secondary Analysis of a Randomized Clinical Trial. JAMA Oncol 6: 519-527, 2020.

13. Foster CC, Couey MA, Kochanny SE, et al.: Immune-related adverse events are associated with improved response, progression-free survival, and overall survival for patients with head and neck cancer receiving immune checkpoint inhibitors. Cancer 127: 4565-4573, 2021.

14. Freeman-Keller M, Kim Y, Cronin H, Richards A, Gibney G and Weber JS: Nivolumab in Resected and Unresectable Metastatic Melanoma: Characteristics of Immune-Related Adverse Events and Association with Outcomes. Clin Cancer Res 22: 886-894, 2016.

15. Fujisawa Y, Yoshino K, Otsuka A, et al.: Retrospective study of advanced melanoma patients treated with ipilimumab after nivolumab: Analysis of 60 Japanese patients. J Dermatol Sci 89: 60-66, 2018.

16. Ge X, Jiang W, Li H, Wu Y, Li X and Cui S: Immune-related adverse events and outcomes among pan-cancer patients receiving immune checkpoint inhibitors: A monocentric real-world observational study. Cancer Med 12: 18491-18502, 2023.

17. Haratani K, Hayashi H, Chiba Y, et al.: Association of Immune-Related Adverse Events With Nivolumab Efficacy in Non-Small-Cell Lung Cancer. JAMA Oncol 4: 374-378, 2018.

18. Hosoya K, Fujimoto D, Morimoto T, et al.: Association Between Early Immune-related Adverse Events and Clinical Outcomes in Patients With Non-Small Cell Lung Cancer Treated With Immune Checkpoint Inhibitors. Clin Lung Cancer 21: e315-e328, 2020.

19. Lee YJ, Kim HT, Won CH, et al.: Characterization and Prognostic Significance of Cutaneous Adverse Events to Anti-Programmed Cell Death-1 Therapy. J Korean Med Sci 34: e186, 2019.

20. Maillet D, Corbaux P, Stelmes JJ, et al.: Association between immune-related adverse events and long-term survival outcomes in patients treated with immune checkpoint inhibitors. Eur J Cancer 132: 61-70, 2020.

21. Min Lee CK, Li S, Tran DC, et al.: Characterization of dermatitis after PD-1/PD-L1 inhibitor therapy and association with multiple oncologic outcomes: A retrospective case-control study. J Am Acad Dermatol 79: 1047-1052, 2018.

22. Morimoto K, Yamada T, Takumi C, et al.: Immune-Related Adverse Events Are Associated With Clinical Benefit in Patients With Non-Small-Cell Lung Cancer Treated With Immunotherapy Plus Chemotherapy: A Retrospective Study. Front Oncol 11: 630136, 2021.

23. Nakamura Y, Kitano S, Takahashi A, et al.: Nivolumab for advanced melanoma: pretreatment prognostic factors and early outcome markers during therapy. Oncotarget 7: 77404-77415, 2016.

24. Nakamura Y, Tanaka R, Asami Y, et al.: Correlation between vitiligo occurrence and clinical benefit in advanced melanoma patients treated with nivolumab: A multi-institutional retrospective study. J Dermatol 44: 117-122, 2017.

25. Nakano E, Takahashi A, Namikawa K, et al.: Correlation between cutaneous adverse events and prognosis in patients with melanoma treated with nivolumab: A single institutional retrospective study. J Dermatol 47: 622-628, 2020.

26. Naqash AR, Ricciuti B, Owen DH, et al.: Outcomes associated with immune-related adverse events in metastatic non-small cell lung cancer treated with nivolumab: a pooled exploratory analysis from a global cohort. Cancer Immunol Immunother 69: 1177-1187, 2020.

27. Nardin C, Jeand'heur A, Bouiller K, et al.: Vitiligo under anti-programmed cell death-1 therapy is associated with increased survival in melanoma patients. J Am Acad Dermatol 82: 770-772, 2020.

28. Nasca V, Barretta F, Corti F, et al.: Association of immune-related adverse events with the outcomes of immune checkpoint inhibitors in patients with dMMR/MSI-H metastatic colorectal cancer. J Immunother Cancer 11, 2023.

29. Nelson CA, Singer S, Chen T, et al.: Bullous pemphigoid after anti-programmed death-1 therapy: A retrospective case-control study evaluating impact on tumor response and survival outcomes. J Am Acad Dermatol 87: 1400-1402, 2022.

30. Ng KYY, Tan SH, Tan JJE, et al.: Impact of Immune-Related Adverse Events on Efficacy of Immune Checkpoint Inhibitors in Patients with Advanced Hepatocellular Carcinoma. Liver Cancer 11: 9-21, 2022.

31. Paderi A, Giorgione R, Giommoni E, et al.: Association between Immune Related Adverse Events and Outcome in Patients with Metastatic Renal Cell Carcinoma Treated with Immune Checkpoint Inhibitors. Cancers (Basel) 13, 2021.

32. Ricciuti B, Genova C, De Giglio A, et al.: Impact of immune-related adverse events on survival in patients with advanced non-small cell lung cancer treated with nivolumab: long-term outcomes from a multi-institutional analysis. J Cancer Res Clin Oncol 145: 479-485, 2019.

33. Sanda GE, Shabto JM, Goyal S, et al.: Immune-Related Adverse Events and Clinical Outcomes in Advanced Urothelial Cancer Patients Treated With Immune Checkpoint Inhibitors. Oncologist, 2023.

34. Sanlorenzo M, Vujic I, Daud A, et al.: Pembrolizumab Cutaneous Adverse Events and Their Association With Disease Progression. JAMA Dermatol 151: 1206-1212, 2015.

35. Shreberk-Hassidim R, Aizenbud L, Lussheimer S, et al.: Dermatological adverse events under programmed cell death-1 inhibitors as a prognostic marker in metastatic melanoma. Dermatol Ther 35: e15747, 2022.

36. Tang K, Seo J, Tiu BC, et al.: Association of Cutaneous Immune-Related Adverse Events With Increased Survival in Patients Treated With Anti-Programmed Cell Death 1 and Anti-Programmed Cell Death Ligand 1 Therapy. JAMA Dermatol 158: 189-193, 2022.

37. Tarhini AA, Kang N, Lee SJ, et al.: Immune adverse events (irAEs) with adjuvant ipilimumab in melanoma, use of immunosuppressants and association with outcome: ECOG-ACRIN E1609 study analysis. J Immunother Cancer 9, 2021.

38. Thompson LL, Nadelmann ER, Blum AE, et al.: Patterns and prognostic significance of cutaneous immune-related adverse events in non-small cell lung cancer. Eur J Cancer 147: 13-16, 2021.

39. Ueda K, Suekane S, Kurose H, et al.: Immune-related adverse events are clinical biomarkers to predict favorable outcomes in advanced renal cell carcinoma treated with nivolumab plus ipilimumab. Jpn J Clin Oncol 52: 479-485, 2022.

40. Wu CE, Yang CK, Peng MT, et al.: The association between immune-related adverse events and survival outcomes in Asian patients with advanced melanoma receiving anti-PD-1 antibodies. BMC Cancer 20: 1018, 2020.

41. Yamazaki N, Kiyohara Y, Uhara H, et al.: Efficacy and safety of nivolumab in Japanese patients with previously untreated advanced melanoma: A phase II study. Cancer Sci 108: 1223-1230, 2017.

42. Yoneda T, Sone T, Koba H, et al.: Long-Term Survival of Patients With Non-Small Cell Lung Cancer Treated With Immune Checkpoint Inhibitor Monotherapy in Real-World Settings. Clin Lung Cancer 23: 467-476, 2022.

43. Zhang S, Tang K, Wan G, et al.: Cutaneous immune-related adverse events are associated with longer overall survival in advanced cancer patients on immune checkpoint inhibitors: A multi-institutional cohort study. J Am Acad Dermatol 88: 1024-1032, 2023.

44. Zhang X, Xu S, Wang J, et al.: Are anti-PD-1-associated immune related adverse events a harbinger of favorable clinical prognosis in patients with gastric cancer? BMC Cancer 22: 1136, 2022.

45. Zhao JJ, Wen XZ, Ding Y, et al.: Association between immune-related adverse events and efficacy of PD-1 inhibitors in Chinese patients with advanced melanoma. Aging (Albany NY) 12: 10663-10675, 2020.
